# Malnutrition, inflammation, progression of vascular calcification and survival: Inter-relationships in hemodialysis patients

**DOI:** 10.1371/journal.pone.0216415

**Published:** 2019-05-02

**Authors:** Sun Ryoung Choi, Young-Ki Lee, A Jin Cho, Hayne Cho Park, Chae Hoon Han, Myung-Jin Choi, Ja-Ryong Koo, Jong-Woo Yoon, Jung Woo Noh

**Affiliations:** 1 Department of Internal Medicine, Hallym University Dongtan Sacred Heart Hospital, Dongtan, Republic of Korea; 2 Department of Internal Medicine, Hallym University Kangnam Sacred Heart Hospital, Seoul, Republic of Korea; 3 Department of Internal Medicine, Hallym University Chuncheon Sacred Heart Hospital, Chuncheon, Republic of Korea; International University of Health and Welfare, School of Medicine, JAPAN

## Abstract

**Background and aims:**

Malnutrition and inflammation are closely linked to vascular calcification (VC), the severity of which correlate with adverse outcome. However, there were few studies on the interplay between malnutrition, inflammation and VC progression, rather than VC presence per se. We aimed to determine the relationship of malnutrition, inflammation, abdominal aortic calcification (AAC) progression with survival in hemodialysis (HD) patients.

**Methods:**

Malnutrition and inflammation were defined as low serum albumin (< 40 g/L) and high hs-CRP (≥ 28.57 nmol/L), respectively. We defined AAC progression as an increase in AAC score using lateral lumbar radiography at both baseline and one year later. Patients were followed up to investigate the impact of AAC progression on all-cause and cardiovascular mortality.

**Results:**

AAC progressed in 54.6% of 97 patients (mean age 58.2±11.7 years, 41.2% men) at 1-year follow-up. Hypoalbuminemia (Odds ratio 3.296; 95% confidence interval 1.178–9.222), hs-CRP (1.561; 1.038–2.348), low LDL-cholesterol (0.976; 0.955–0.996), and the presence of baseline AAC (10.136; 3.173–32.386) were significant risk factors for AAC progression. During the mean follow-up period of 5.9 years, 38(39.2%) patients died and 27(71.0%) of them died of cardiovascular disease. Multivariate Cox regression analysis adjusted for old age, diabetes, cardiovascular history, and hypoalbuminemia determined that AAC progression was an independent predictor of all-cause mortality (2.294; 1.054–4.994).

**Conclusions:**

Malnutrition and inflammation were significantly associated with AAC progression. AAC progression is more informative than AAC presence at a given time-point as a predictor of all-cause mortality in patients on maintenance HD.

## Introduction

Vascular calcification (VC) is prevalent among hemodialysis (HD) patients, and its extent and severity correlated with cardiovascular morbidity and even mortality [1–3]. The main vascular lesions in HD patients are not atherosclerotic plaques but partly medial calcification of the arteries [4], which is associated with nontraditional risk factors such as disturbed mineral metabolism, inflammation, malnutrition, and oxidative stress [5, 6]. A critical inducer of disordered mineral and bone metabolism catalyzes the osteochondrogenic conversion of vascular smooth muscle cells (VSMCs), which plays major role in the development of VC [7]. Despite growth in knowledge, the predictors for VC are not fully understood. Furthermore, correcting a disturbed mineral metabolism also fails to substantially improve clinical outcomes [8] and controlling the relentless progression of VC has become even more challenging [9].

Current evidence suggests that malnutrition and inflammation are closely interrelated and work together to promote VC [10]. Persistent low grade systemic inflammation increases levels of circulating inflammatory markers such as C-reactive protein (CRP), interleukin-6, and tumor necrosis factor-α [11, 12]. Hypoalbuminemia could be a consequence of an inflammation-mediated inability of HD patients to decrease the albumin fractional catabolic rate during protein restriction although it was presumed to arise primarily from malnutrition [13]. It was reported that malnutrition assessed by serum albumin level was best predicted by hs-CRP level[14].

While most studies have focused on the presence or absence of VC in HD patients, there were very few studies which have focused on the association between malnutrition, inflammation and VC progression in HD patients. Many studies which have observed significant VCs on plain radiographs can be a vital source of information for mortality in HD patients [15]. Recently, the progression rather than presence of VC has been recognized as a more critical risk factor [2, 3]. The present study aimed to investigate the risk factors encompassing malnutrition, inflammation and mineral metabolism implicated in abdominal aortic calcification (AAC) progression, and evaluate the impact of AAC progression on cardiovascular outcome and survival in patients on maintenance HD.

## Materials and methods

### Study population

This prospective observational study and was approved by the Institutional Review Board of Hallym University Kangnam Sacred Hospital in Korea (IRB no. 2010-05-33). We received written informed consent from the patients. A total of 156 chronic HD patients were enrolled from the dialysis unit of Hallym University Kangnam and Chuncheon Sacred Hospital in January 2011. We enrolled patients aged > 18 years who were on bicarbonate (30~40mmol/L)-based HD with a calcium concentration of 1.25~1.5 mmol/L and no phosphorus scheduled thrice weekly for 4 hours per session. The exclusion criteria were: (1) significant co-morbidities such as malignancy that were estimated to reduce life expectancy, (2) clinical evidence of either acute infectious or inflammatory diseases for at least 4 weeks before enrollment, (3) a prior history of peritoneal dialysis or kidney transplantation, and (4) to undergo lateral lumbar radiography in the standing position. From the 156 patients at study enrollment, 43 were lost to follow-up. At 1 year of follow-up, 16 patients for whom data were missing were excluded. The remaining 97 patients were examined for the factors affecting AAC progression and followed up until death or August 2018 to evaluate the association between AAC progression and mortality.

### Clinical and biochemical characteristics

Baseline demographic and clinical characteristics were evaluated at study enrollment. Old age was defined as age ≥ 65 years. Cardiovascular disease includes coronary artery, cerebrovascular and peripheral vascular diseases. Blood samples were obtained before the dialysis session under fasting conditions to measure various markers using standard techniques. The measured serum calcium level was corrected using the following formula when hypoalbuminemia was present: total serum calcium + 0.8 × (4.0 –measured serum albumin). Overweight and obesity was defined as a body mass index (BMI) of 25–29.9 kg/m^2^ and >30 kg/m^2^, respectively [16]. Kt/V was calculated using the logarithmic estimate of the Daugirdas method [17].

### Assessment of AAC

AAC score was calculated using a previously validated method by Kauppila LI et al [18]. At baseline and 1 year later, lateral lumbar radiographs were obtained from a fixed distance of 100 cm using standard radiographic equipment with the subjects in a standing position. The severity of the anterior and posterior aortic calcification was graded individually on a 0–3 scale for each lumbar segment (L1–L4) and the results were summarized to develop the AAC score (range 0–24). Progression of AAC was defined as an increase in AAC score after 1 year of follow-up. The baseline and follow-up films were examined in pairs. All films were scored by two independent observers without knowledge of the subjects’ clinical background.

To assess intra-observer correlation in interpreting the AAC score, all abdominal radiographs were re-interpreted independently by the same doctors after a 1-month period, without knowing clinical data and previous scores. Then, correlation was calculated comparing the present and the previous findings. The Intraclass correlation coefficient (ICC) analysis between two examiners using SPSS version 18.0 (SPSS, Inc., Chicago, IL, USA) was 0.91. This value can be said to be very reliable between the examiners.

### Definition of malnutiriton and inflammation

Hypoalbuminemia (serum albumin < 40 g/L) [19] and a high level of high-sensitivity CRP (hs-CRP ≥ 28.57 nmol/L) [20] were used as markers of malnutrition and inflammation, respectively. Patients were divided into three groups according to the number of malnutrition and inflammatory markers.

### Statistical analysis

Summary statistics are expressed as means ± standard deviations or median for continuous variables and as frequencies or percentages for categorical variables. Continuous variables were compared using Student’s t-test for two groups and one-way analysis of variance for three groups or the Mann-Whitney U test for a non-parametric test and categorical variables were compared using the Chi-square test or Fisher’s exact test as appropriate. Uni- and multivariate logistic regression analysis was performed to investigate the determinants of AAC progression and presence. The Kaplan-Meier method for survival analysis and log-rank test for comparison of survival rate differences between patients with and without AAC progression as well as AAC presence were used. Cox proportional hazard regression models were constructed to evaluate the influence of AAC progression and presence on mortality. All tests were performed using SPSS. A *p*-value of less than 0.05 was considered statistically significant.

## Results

### Baseline characterisrics according to ACC progression

**Fig 1:**
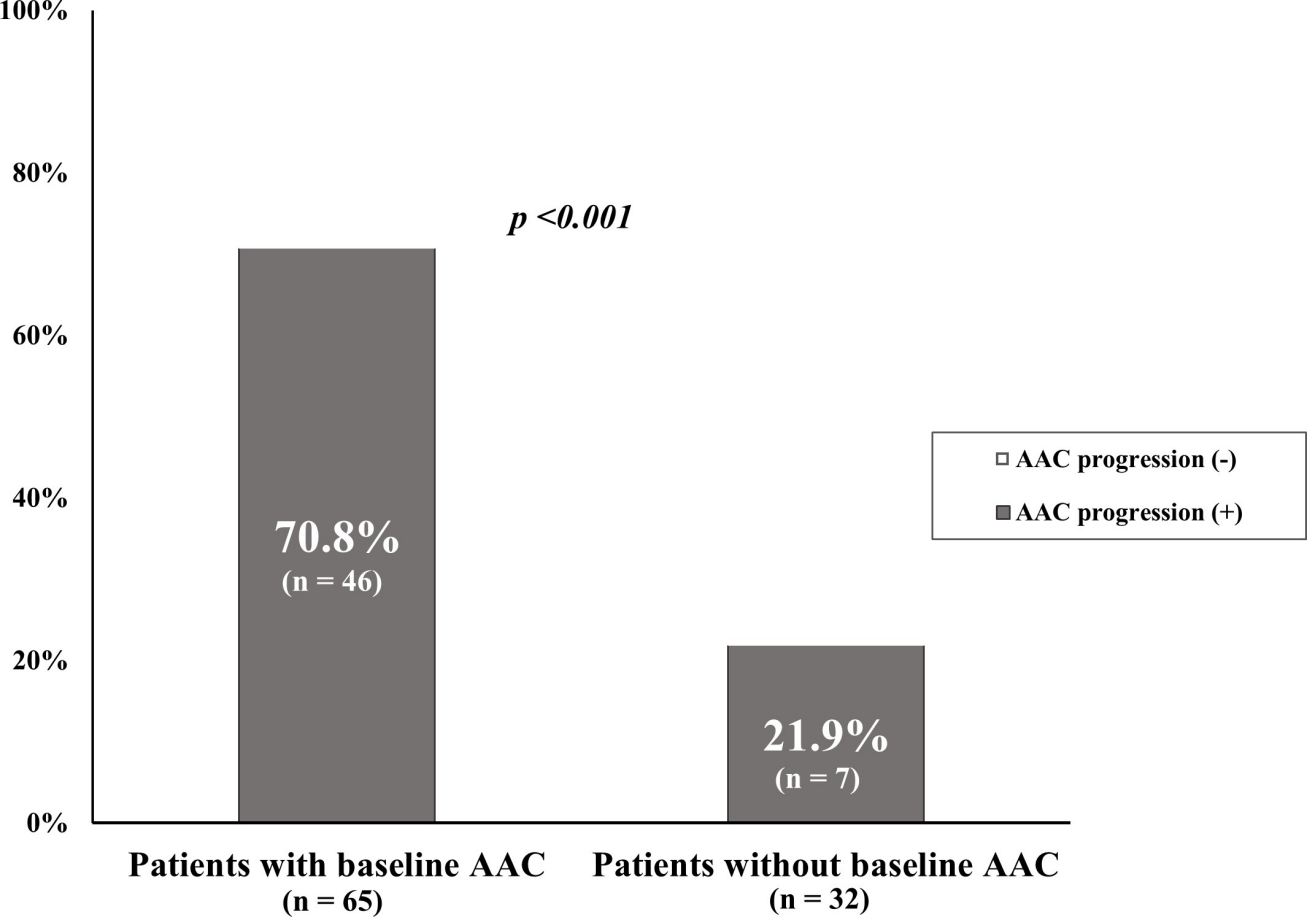
Portion of patients with and without AAC progression according to presence or absence of baseline AAC. AAC, abdominal aortic calcification.

The mean age of patients was 58.2 ± 11.7 (range, 33–83) years. The proportion of patients with presence of AAC at baseline were 67.0% and mean baseline AAC score was 4.5 ± 5.4 (range, 0–22). The proportion of the patients with AAC progression 1 year later were 54.6% and mean AAC score was 10.1 ± 6.3 (range, 0–24). There was a significant difference of AAC progression rate between the patients with baseline AAC and those without baseline AAC (70.8% vs. 21.9%, *p* <0.001, Fig 1). Patients with AAC progression were more likely to be older; have a have cardiovascular history; have lower levels of albumin, total cholesterol, and low- density lipoprotein(LDL) cholesterol; and have higher levels of hs-CRP and intact parathyroid hormon(iPTH) than those without progression (Table 1). Both the prevalence of baseline AAC (86.8% vs. 43.2%, *p* <0.001) and baseline AAC score (6.3 ± 5.9 vs. 2.3 ± 3.6, *p* = 0.001) were higher in patients with AAC progression than those without. The mean follow-up AAC score in patients with AAC progression was 10.0 ± 6.3, an increase of 3.6 ± 2.7 from 1 year prior.

**Table 1:** Clinical characteristics and laboratory results according to AAC progression.

	Total n = 97	AAC progression (+) n = 53	AAC progression (-) n = 44	*p* value
Demographic data				
Age, years	58.2 ± 11.7	61.6 ± 11.0	54.0 ± 11.3	0.001
Male, n (%)	40 (41.2)	27 (50.9)	13 (29.5)	0.033
Comorbidities, n (%)				
Cardiovascular disease	39 (40.2)	28 (52.8)	11 (25.0)	0.005
Diabetes Mellitus	57 (58.8)	33 (62.3)	24 (54.5)	0.442
Hemodialysis duration, years	4.6 ± 4.4	4.9 ± 4.8	4.3 ± 3.9	0.551
BMI, kg/m^2^	22.2 ± 3.2	22.2 ± 3.3	22.1 ± 3.1	0.771
Kt/V	1.5 ± 0.2	1.4 ± 0.2	1.5 ± 0.2	0.147
Current medication, n (%)				
Aspirin	80 (82.5)	42 (79.2)	38 (86.4)	0.359
Statin	17 (17.5)	14 (26.4)	3 (6.8)	0.010
Vitamin D analogues	25 (25.8)	15 (28.3)	10 (22.7)	0.532
Phosphate binder	59 (60.8)	34 (64.2)	25 (56.8)	0.016
Laboratory data				
Albumin, g/L	39.14 ± 4.98	38.15 ± 5.79	40.3 ± 3.47	0.030
hs-CRP, nmol/L	19.53 ± 20.46	24.23 ± 25.10	13.86 ± 10.61	0.012
Total cholesterol, mmol/L	3.84 ± 0.90	3.63 ± 0.81	4.08 ± 0.96	0.014
Triglyceride, mmol/L	1.30 ± 0.94	1.35 ± 0.65	1.24 ± 1.20	0.564
HDL-cholesterol, mmol/L	0.96 ± 0.26	0.92 ± 0.22	1.01 ± 0.30	0.110
LDL-cholesterol, mmol/L	2.15 ± 0.73	1.97 ± 0.69	2.37 ± 0.72	0.006
Calcium, mmol/L	2.14 ± 0.22	2.11 ± 0.23	2.18 ± 0.19	0.156
Phosphate, mmol/L	1.59 ± 0.46	1.58 ± 0.50	1.60 ± 0.42	0.877
iPTH, ng/L	205.4 ± 182.7	241.5 ± 212.8	161.8 ± 127.4	0.032

AAC, Abdominal aortic calcification, BMI = body mass index; hs-CRP = high-sensitivity C-reactive protein; HDL = high-density lipoprotein; LDL = low-density lipoprotein; iPTH = intact parathyroid hormone.

### AAC progression according to malnutrition and inflammation markers

The rate of hypoalbuminemia and a high hs-CRP level in patients with any 1 marker was 81.4% and 18.6%, respectively. The incidence of patients older than 65 years, diabetes mellitus (DM), and a history of previous cardiovascular disease was significantly lower in patients without any markers (Table 2). Serum phosphate and high-density lipoprotein (HDL)-cholesterol levels tended to be higher in patients without any markers than in patients with any marker, while, the proportion of patients with a BMI ≥ 25 kg/m^2^ tended to increase as the number of malnutrition and inflammation markers increase (0% vs. 13.9% vs. 76.9%, *p* <0.001).

**Table 2:** Clinical characteristics and laboratory results according to the number of malnutrition and inflammation markers.

Variables	Number of malnutrition and inflammation markers n = 97			*p* value
	0 n = 41	1 n = 43	2 n = 13	
Demographic data				
Age, years	54.2 ± 9.9	61.2 ± 11.7	60.9 ± 13.9	0.014
Male, n (%)	14 (34.1)	18 (41.9)	8 (61.5)	0.216
Comorbidities, n (%)				
Cardiovascular disease	11 (26.8)	19 (44.2)	9 (69.2)	0.019
Diabetes Mellitus	17 (41.5)	31 (72.1)	9 (69.2)	0.012
Hemodialysis duration, years	5.4 ± 5.0	4.4 ± 4.1	2.9 ± 2.7	0.213
BMI, kg/m^2^	21.2 ± 2.1	21.9 ± 3.3	26.1 ± 2.8	<0.001
Laboratory data				
Albumin, g/L	42.78 ± 4.28	36.74 ± 3.84	35.61 ± 2.50	<0.001
hs-CRP, nmol/L	11.47 ± 7.11	18.39 ± 15.86	48.68 ± 33.96	<0.001
Total cholesterol, mmol/L	3.95 ± 0.92	3.77 ± 0.90	3.73 ± 0.86	0.600
Triglyceride, mmol/L	1.08 ± 0.67	1.44 ± 1.17	1.55 ± 0.67	0.131
HDL-cholesterol, mmol/L	1.06 ± 0.29	0.89 ± 0.24	0.87 ± 0.15	0.006
LDL-cholesterol, mmol/L	2.26 ± 0.86	2.09 ± 0.62	2.03 ± 0.61	0.472
Calcium, mmol/L	2.20 ± 0.20	2.09 ± 0.22	2.15 ± 0.24	0.056
Phosphate, mmol/L	1.76 ± 0.45	1.51 ± 0.46	1.30 ± 0.30	0.003
iPTH, ng/L	194.0 ± 151.4	226.8 ± 224.7	170.0 ± 102.9	0.544

BMI = body mass index; hs-CRP = high-sensitivity C-reactive protein; HDL = high-density lipoprotein; LDL = low-density lipoprotein; iPTH = intact parathyroid hormone, AAC, Abdominal aortic calcification

**Fig 2:**
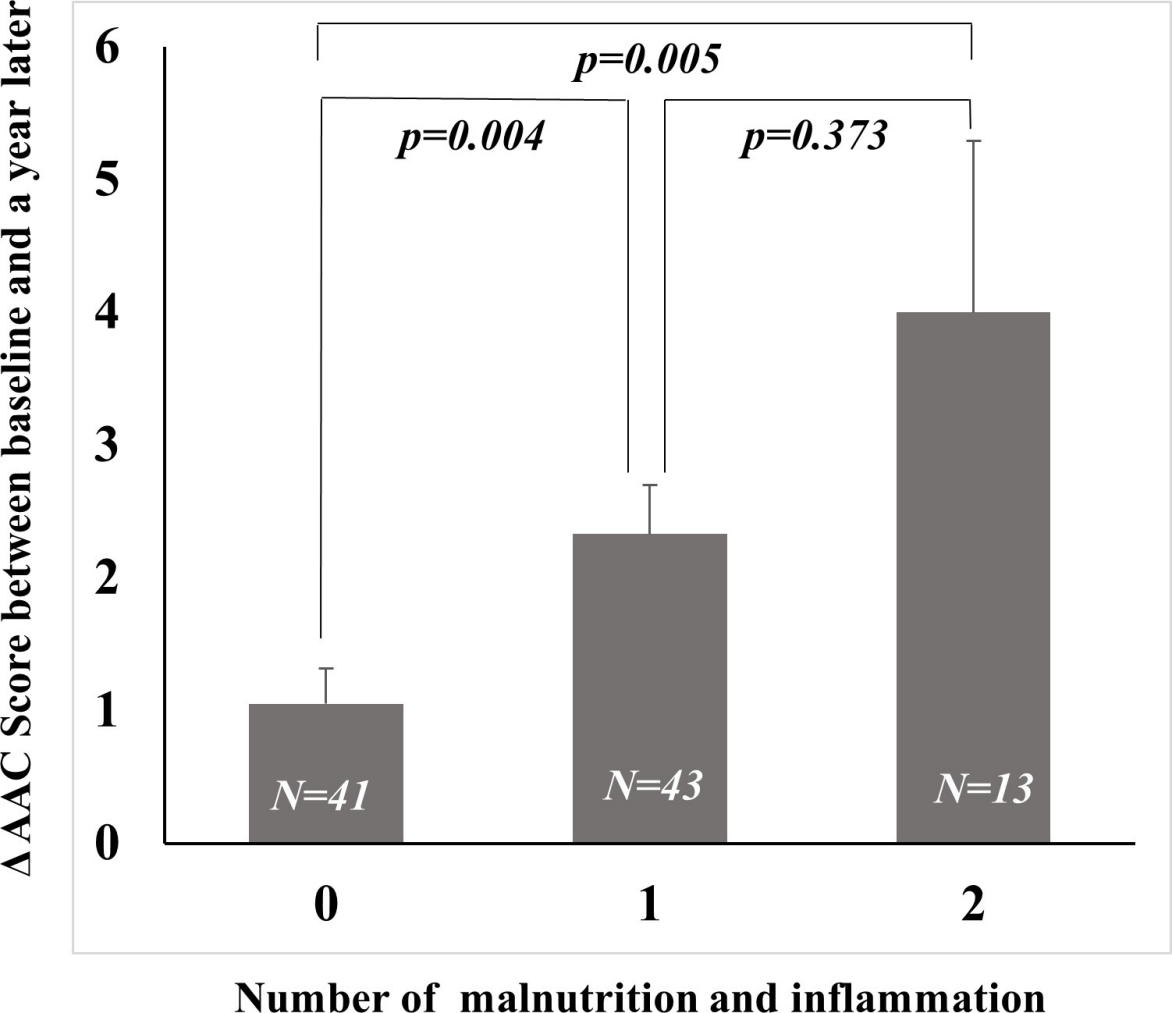
Changes in AAC score according to the number of malnutrition and inflammation markers. Median AAC scores increased from 1, 3, and 5 to 3, 7, and 8 in patients without any markers, with any 1 marker, and with 2 markers, respectively.

The incidence of baseline AAC was 56.1%, 74.4% and 76.9% in patients without any markers, with any 1 marker, and with 2 markers, respectively, with no significant difference (*p* = 0.146). The patients with both malnutrition and inflammation markers had higher proportion of AAC progression within a year (34.1% (0 marker) vs. 67.4% (1 marker) and 76.9% (2 markers), *p* = 0.002). The degree of AAC progression was higher in those with both malnutrition and inflammation markers compared with those with lesser number of markers in Fig 2 (Δ AAC 1.0 ± 1.6 (0 marker) vs. 2.3 ± 2.4 (1 marker) vs. 4.0 ± 4.6 (2 markers), *p* = 0.003). The mean AAC scores significantly increased at 1-year follow-up in patients without any markers, with any 1 marker, and with 2 markers, respectively (Table 3).

**Table 3:** AAC score after 1 year later according to the number of malnutrition and inflammation markers.

Variables	Number of malnutrition and inflammation markers n = 97			*p* value
	0 n = 41	1 n = 43	2 n = 13	
AAC at baseline	2.93 ± 3.79	5.53 ± 5.83	6.31 ± 7.15	0.037
AAC after 1 year later	3.98 ± 4.53	7.86 ± 6.46	10.31 ± 9.24	0.004

AAC, Abdominal aortic calcification

### Risk factors for AAC progression

We performed logistic regression analysis to identify risk factors associated with AAC progression. In univariate analysis, hypoalbuminemia, hs-CRP, low LDL-cholesterol, iPTH, and the presence of baseline AAC were significantly associated with AAC progression. However, hypoalbuminemia (odds ratio, 3.296; 95% confidence interval, 1.178–9.222), hs-CRP (1.561; 1.038–2.348), AAC presence at baseline (10.136; 3.173–32.386), and low LDL-cholesterol level (0.976; 0.955–0.996) were significant independent predictors of AAC progression (Table 4) except for iPTH in multivariate analysis.

**Table 4:** Risk factors for abdominal aortic calcification progression.

Variables	Univariate		Multivariate	
	Odds ratio (95% CI)	*p* value	Odds ratio (95% CI)	*p* value
Hypoalbuminemia (yes vs. no)	3.835 (1.644–8.946)	0.002	3.296 (1.178–9.222)	0.023
hs-CRP, nmol/L	1.546 (1.095–2.182)	0.013	1.561 (1.038–2.348)	0.032
LDL-cholesterol, mmol/L	0.979 (0.963–0.995)	0.009	0.976 (0.955–0.996)	0.020
iPTH, ng/L	1.003 (1.000–1.006)	0.040		
Baseline AAC (yes vs. no)	8.647 (3.200–23.364)	< 0.001	10.136 (3.173–32.386)	<0.001

hs-CRP = high-sensitivity C-reactive protein; LDL = low-density lipoprotein; iPTH = intact parathyroid hormone.

### AAC progression and predictors of all-cause mortality

**Fig 3:**
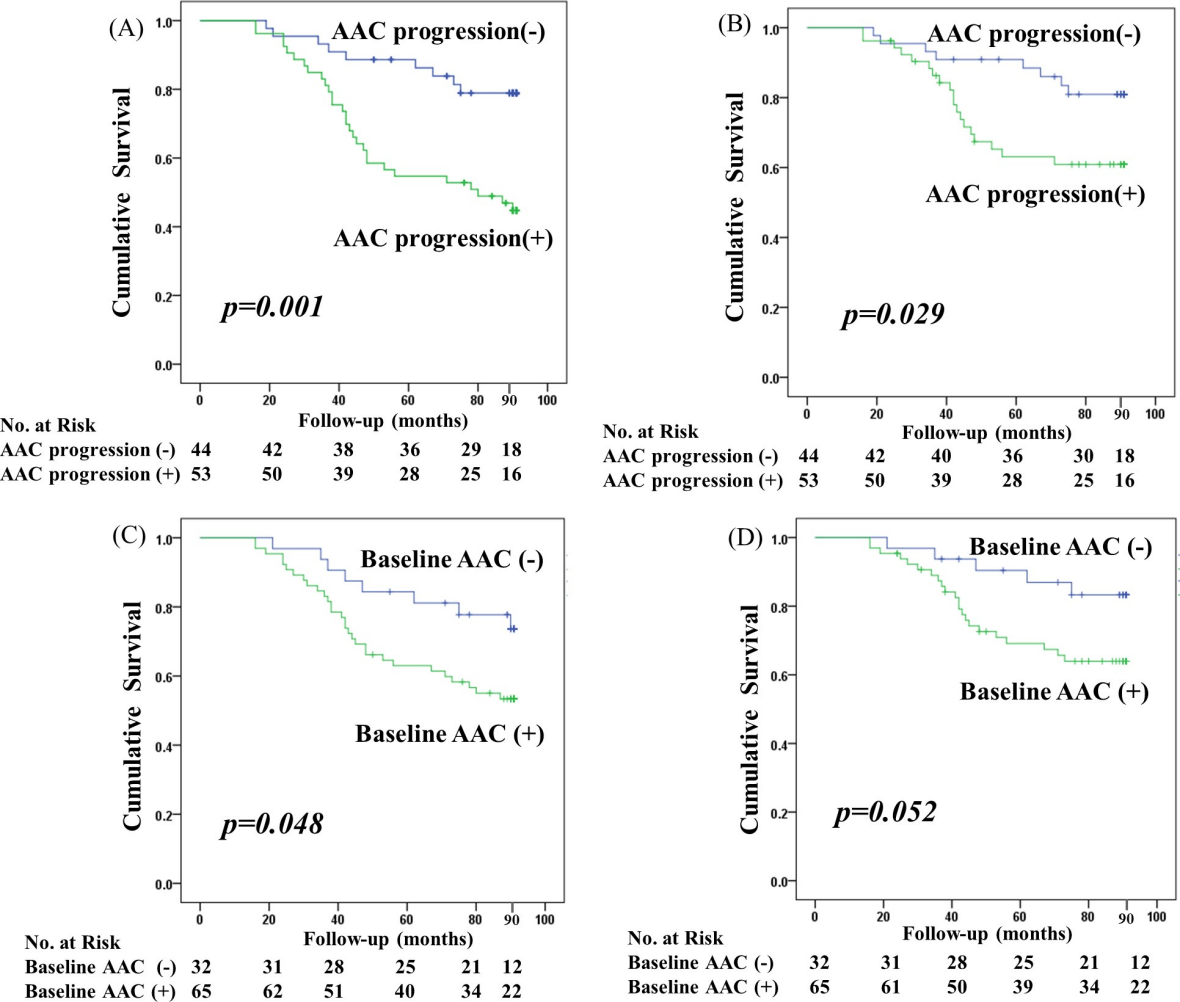
Survival curves for all-cause and cardiovascular mortality. (A) All-cause mortality according to AAC progression. (B) Cardiovascular mortality according to AAC progression. (C) All-cause mortality according to baseline AAC. (D) Cardiovascular mortality according to baseline AAC. The 7.5-year overall survival was significantly lower in patients with AAC progression (45.3% vs. 79.5%) as well as baseline AAC (53.8% vs. 75.0%) than in those without.

Mean follow-up was 5.9 years (interquartile range, 1.3–7.5; median, 7.5). Survival analysis showed that all-cause and cardiovascular mortality rate were significantly higher in patients with AAC progression than in those without (log-rank test, *p* = 0.001 and 0.029, respectively). Patients with baseline AAC were also at significantly higher risk for all-cause (log-rank test, *p* = 0.048), but not cardiovascular (*p* = 0.052) mortality (Fig 3). Multivariate Cox proportional hazard analysis adjusted for old age, DM, history of cardiovascular disease, hypoalbuminemia, hs-CRP level, and the presence of baseline AAC determined that AAC progression was significantly associated with inferior survival. Old age and DM were also independent risk factors for all-cause mortality (Table 5). But AAC presence at baseline was not an independent predictor of all-cause mortality in multivariate Cox regression analysis. Overall, 38 deaths were identified in this study period. Cardiovascular death (n = 27, 71.0%) was the most common cause in patients with (19/29, 65.5%) and without (8/9, 88.8%) AAC progression.

**Table 5:** Predictors of all-cause mortality.

	Univariate			Multivariate		
	Odds ratio	95% CI	*p* value	Odds ratio	95% CI	*p* value
Old age (yes vs. no)	4.577	2.389–8.771	<0.001	3.651	1.859–7.173	<0.001
Diabetes Mellitus (yes vs. no)	3.882	1.707–8.829	0.001	3.465	1.515–7.926	0.003
Cardiovascular history (yes vs. no)	2.330	1.220–4.450	0.010			
Hypoalbuminemia (yes vs. no)	2.613	1.317–5.183	0.006			
hs-CRP, nmol/L	1.091	0.983–1.211	0.100			
AAC presence (yes vs. no)	2.155	0.988–4.703	0.054			
AAC progression (yes vs. no)	3.319	1.569–7.022	0.002	2.294	1.054–4.994	0.037

hs-CRP = high-sensitivity C-reactive protein

## Discussion

The findings of this study are as follows: in patients on maintenance HD, (1) malnutrition and inflammation were significant risk factors for AAC progression; (2) AAC progression was an independent predictor for all-cause mortality; and (3) AAC progression was more informative than AAC presence as a predictor of all-cause mortality.

One strength of this study is that we reported relatively long-term outcomes. Long-term follow-up was available for 97 patients recruited at two dialysis centers. Another strength is that there were very few studies on the association between malnutrition, inflammation, and VC progression, rather than VC presence per se. Among most previous studies of the pathophysiologic link between malnutrition, inflammation and atherosclerosis, the definition of atherosclerosis has been described simply using VC presence without quantification or cardiovascular history [21–23]. To the best of our knowledge, there is only a single data similar to our study. Okamoto et al.[24] assessed an AAC index for HD patients from their annual abdominal CT and suggested that poor nutritional status was independently associated with rapid ΔAAC index progression. Our data was not limited to a higher ΔAAC progression but included all patients with AAC progression. Moreover, AAC progression was further refined according to the number of malnutrition and inflammation factors.

Increased serum hs-CRP and decreased albumin levels were independent predictors of AAC progression in the present study. Ishimura et al. indicated that a high CRP level was significantly related with VC in both the aorta and the hand arteries on plain radiographs in dialysis patients [25]. Desjardins et al. reported that plasma interleukin-6 was significantly associated with aortic stiffness in dialysis patients [26]. The role of inflammation as a risk factor for malnutrition has been recognized and it was reported that elevated CRP levels could inhibit albumin synthesis in HD patients [27]. Inflammatory cytokines enhance VC through induction of osteogenic phenotype of VSMCs by inhibiting VSMC-specific genes and stimulating osteoblastic genes[28–30]. In dialysis patients, anti-calcific molecule such as fetuin-A may be decreased and dysfunctional. There has been growing interest in fetuin-A as a potent inhibitor for VC progression and a protector of atherosclerosis[31]. Fetuin-A, a hepatic glycoprotein in the circulation with affinity towards hydroxyapatite, was inversely correlated with inflammatory markers, and positively associated with nutritional indicators such as serum albumin and pre-albumin level [32].

Contrary to our expectations, the trend in changes from 0 to the presence of 2 malnutrition and inflammatory markers showed an increase with BMI. The prevalence of overweight patients was significantly higher among patients with both inflammation and malnutrition markers than in patients without any markers. Adipose tissue, especially visceral adiposity, is a source of high cytokine levels among dialysis patients [33]. The second explanation is the high incidence of DM and overweight among dialysis patients [34]. They may stay overweight for the long term. Patients without any inflammation and malnutrition markers showed that the lowest prevalence of DM and mean BMI in our study. This is contrary to the obesity paradox, which indicates to better outcomes with higher BMI and is quite a consistent finding in dialysis patients [35]. Moriyama et al. showed that BMI was correlated with volume of visceral fat using a computed tomography (CT) scan, and that chronic HD patients with a larger volume of visceral fat showed significantly lower HDL-cholesterol levels, increased CRP levels and severe aortic calcification [34]. In addition, Delgado et al. demonstrated that waist circumference was associated with CRP and inversely associated with albumin level [36]. They regarded visceral fat as an indicator of chronic inflammation. We did not conduct body composition analyses and could only assume the cause of overweight was excess visceral fat or fluid retention due to their poor outcomes. The 7.5-year survival rate of patients with both inflammation and malnutrition markers in this study was only 38.5%.

HDL-cholesterol level was significantly decreased as the number of inflammation and malnutrition markers increased; with this result, reduced LDL-cholesterol level was an independent risk factor for AAC progression in our study. Chronic inflammation in dialysis patients is also associated with dyslipidemia, which is characterized by decreasing total, LDL, and HDL-cholesterol and increasing triglyceride levels [37]. HDL-cholesterol is both decreased and dysfunctional; therefore, it may lose its anti-inflammatory and vasoprotective properties[38]. Reverse epidemiology has been noted in dialysis patients with lower cholesterol levels being associated with higher mortality rates, possibly reflecting the profound malnutrition and inflammation present in dialysis patients [34].

The development of VC in dialysis patients is closely associated with dysregulation of mineral metabolism including long-term elevations of serum phosphate. There were no significant differences between phosphate level and the portion of hyperphosphatemia between patients with and those without AAC progression in this study. This is different from the generally known key role of serum phosphate, which can be explained as follows. First, serum levels of phosphate were in the normal range and there was no statistically significant difference between two groups. Second, calcium-free phosphate binder was used more frequently in patients with AAC progression than those without AAC progression. Lastly, malnutrition and chronic inflammation are presumed to accelerate progression of VC despite of lower level of phosphate in patients who had more than one marker than those without both malnutrition and inflammation marker.

We determined AAC using lateral lumbar radiographs. Kidney Disease: Improving Global Outcomes (KDIGO) guidelines recommend that a lateral abdominal radiography should be performed to assess VC in dialysis patients [39]. Most previous studies focused on the presence of aortic arch calcification, coronary or cardiac valve calcification using chest X-ray or CT scan. Few studies have analyzed the effects of the progression of AAC assessed by lateral lumbar radiography among HD patients. Although a CT scan is the most sensitive and accurate technique for evaluating VC [40], we assumed that this semi-quantitative method also offers acceptable sensitivity and specificity as a useful alternative to a CT scan [18]. AAC in patients on chronic HD reflects the severity of atherosclerosis and correlate with calcification at other sites [41]. Moreover, this method is widely available and inexpensive.

The presence of baseline AAC was one of the most important determinants of AAC progression in the present study. Block et al. demonstrated that patients with evidences of coronary calcification at baseline exhibited significant progression within 6 months of starting HD despite the control of laboratory parameters, whereas, patients without baseline calcification showed little development over 18 months [42]. Sigrist et al. stated that patients with preexisting VC of the superficial femoral artery exhibited significantly increased calcification over 24 months [43]. Recent studies reported that 26–78% of dialysis patients had various degrees of aortic arch calcification on plain radiographs, and 34–60% had progression after 1–5 years of follow-up [44]. Unfortunately, there were no correctable causes for AAC presence in our study, including age and hemodialysis vintage. In subgroup analysis, patients were further divided into 4 groups based on the existence of baseline AAC and progression of AAC. Patients with AAC development had a significantly lower survival rate than those without among patients without preexisting AAC (42.9% vs. 84.0%, *p* = 0.026). These results suggest that considering risk factors for AAC progression can help improve outcomes.

The presence of VC has been shown to be associated with the survival of patients on chronic HD regardless of the methods used to assess VC [45, 46]. Studies about VC and mortality in HD patients have yielded conflicting results. The progression of VC has been recognized as a more important predictor than the presence of VC [2, 3]. Meanwhile, others found that the progression and presence of VC were independently associated with adverse outcomes [47]. In the present study, a multivariate Cox proportional hazard model determined that AAC progression was an independent predictor of all-cause mortality. However, AAC presence was not a statistically significant risk factor although Kaplan-Meier survival curves showed that both the progression of AAC and the presence of baseline AAC were significantly associated with increased all-cause mortality. We think that AAC presence is also an important factor determining clinical outcome, and this result was caused by the relative lack of influence on all-cause mortality compared to other factors.

### Limitations

Our study has several limitations. First, the sample size was small. Second, using plain radiographs to evaluate the presence of AAC may miss some subtle calcifications and changes. Third, the time interval between the AAC measurements may be short, and a longer follow-up period would have yielded more informative result in terms of AAC progression. However, similarly to the present study, previous reports also showed significant progression of VC in less than a year. In addition, regular measures of AAC at regular intervals during the study period may be more advisable. Fourth, we did not perform bioimpedance spectroscopy or waist circumference measurements; thus, we could only guess the cause of overweight or obesity using BMI. Therefore, further studies are necessary to confirm these results.

## Conclusion

This study demonstrated that malnutrition and inflammation were significant risk factors for AAC progression. In addition, other statistically significant findings related with AAC progression were also associated with the chronic inflammatory status of HD patients. Studies of the effectiveness of an anti-inflammatory or nutritional intervention on VC progression should be considered. We confirmed that AAC progression was an independent risk factor for all-cause mortality in HD patients, and although AAC presence itself is also an important risk factor for adverse outcomes, we suggest that AAC progression is more informative than AAC presence at a given time-point as a predictor of all-cause mortality in patients on maintenance HD.
